# Functionalized *β*-Cyclodextrin Immobilized on Ag-Embedded Silica Nanoparticles as a Drug Carrier

**DOI:** 10.3390/ijms20020315

**Published:** 2019-01-14

**Authors:** Eun Ji Kang, Yu Mi Baek, Eunil Hahm, Sang Hun Lee, Xuan-Hung Pham, Mi Suk Noh, Dong-Eun Kim, Bong-Hyun Jun

**Affiliations:** 1 Department of Bioscience and Biotechnology, Konkuk University, Seoul 05029, Korea; ejkang@konkuk.ac.kr (E.J.K.); undine1213@naver.com (Y.M.B.); greenice@konkuk.ac.kr (E.H.); shlee.ucb@gmail.com (S.H.L.); phamricky@gmail.com (X.-H.P.); kimde@konkuk.ac.kr (D.-E.K.); 2 Bio-Health Convergence Institute, Korea Testing Certification, Gunpo 15809, Korea; pourlady@ktc.re.kr

**Keywords:** cyclodextrin, doxorubicin (DOX), drug delivery

## Abstract

Cyclodextrins (CDs) have beneficial characteristics for drug delivery, including hydrophobic interior surfaces. Nanocarriers with *β*-CD ligands have been prepared with simple surface modifications as drug delivery vehicles. In this study, we synthesized *β*-CD derivatives on an Ag-embedded silica nanoparticle (NP) (SiO_2_@Ag NP) structure to load and release doxorubicin (DOX). Cysteinyl-*β*-CD and ethylenediamine-*β*-CD (EDA-*β*-CD) were immobilized on the surface of SiO_2_@Ag NPs, as confirmed by transmission electron microscopy (TEM), ultraviolet-visible (UV-Vis) spectrophotometry, and Fourier transform infrared (FTIR) spectroscopy. DOX was introduced into the *β*-CD on the SiO_2_@Ag NPs and then successfully released. Neither cysteinyl-*β*-CD and EDA-*β*-CD showed cytotoxicity, while DOX-loaded cysteinyl-*β*-CD and EDA-*β*-CD showed a significant decrease in cell viability in cancer cells. The SiO_2_@Ag NPs with *β*-CD provide a strategy for designing a nanocarrier that can deliver a drug with controlled release from modified chemical types.

## 1. Introduction

Nanomaterial-based carriers have been widely studied as transport vehicles for various substances, such as drugs, due to their ability to increase local accessibility to the target and enhance bioavailability [1,2,3,4]. It is important to select a suitable nanoparticle (NP) for the fabrication of a nanocarrier as well as a ligand, which will be immobilized on the NP, to capture and release the target drug.

In *β*-cyclodextrin (*β*-CD), the inner cavity is hydrophobic, and the outside is hydrophilic, which is beneficial for incorporating hydrophobic materials into the cavity. The formation of an inclusion complex between *β*-CD and hydrophobic materials can result in the dissolution of insoluble materials in water [5,6,7]. A variety of studies in the food, pharmaceutical, medical, and cosmetic industries have evaluated the complexation ability of *β*-CD [8,9,10]. In the medical field, the inclusion complex between *β*-CD and doxorubicin (DOX) was first reported in the 1990s [11], and subsequent studies have examined its complex-forming ability [12,13,14]. Surface modification is essential for the introduction of *β*-CD as a stable ligand onto NPs (unpublished). In addition, the affinity and materials that can be captured differ depending on the kind of *β*-CD functional group. Moreover, little is known about the amounts of loaded and released DOX using *β*-CD.

Metal-embedded silica NPs (SiO_2_ NPs) have been prepared for various applications and provide several key advantages over other NPs, such as the potential for plasmon tuning for deep tissue imaging [15] and photothermal therapy [16], the strong plasmonic property for sensitive detection [17,18,19,20,21,22], and easy handling and surface modification [17]. Moreover, metal NPs have affinity to ligands with unshared electron pairs, such as thiol and amine groups [18,23]. Using the SiO_2_@Ag NP structure, a target material can be detected by introducing *β*-CD, which is used as a capture ligand. Functionalized *β*-CD included amine (ethylenediamine) and thiol (cysteinyl) group was introduced on the metal surface [24,25]. Detection using the assembled structure and *β*-CD has been reported, but subsequent analyses of drug delivery are lacking. In addition, drug release using functionalized *β*-CD has not been studied.

In this study, we used SiO_2_@Ag NPs coated with cysteinyl-*β*-CD and ethylenediamine (EDA)-*β*-CD as ligands to capture DOX. The rates of loaded and released DOX depended on the kind of *β*-CD. The drug release kinetics differed depending on the kind of immobilized *β*-CD on the SiO_2_@Ag NPs. In addition, we treated breast cancer cells with DOX-loaded SiO_2_@Ag@*β*-CD NPs to assess the cytotoxicity of DOX-loaded cysteinyl-*β*-CD and EDA-*β*-CD. Our results suggest that *β*-CD derivatives could be used for drug capture and release, and cysteinyl-*β*-CD might be useful as a nanocarrier in drug delivery systems.

## 2. Results and Discussion

**Figure 1:**
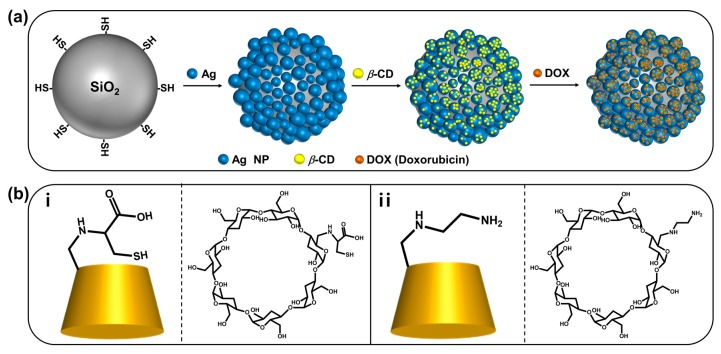
(**a**) Procedure for synthesizing SiO_2_@Ag NPs and introducing *β*-CD derivatives and DOX. (**b**) Chemical structures and illustration of (i) cysteinyl-*β*-CD and (ii) EDA-*β*-CD.

As illustrated in Figure 1a, SiO_2_@Ag NPs, which have the advantage of facile handling and surface modifications, were used to immobilize *β*-CD while maintaining the SiO_2_@Ag NP structure. Then, the functionalized *β*-CD was immobilized on SiO_2_@Ag NPs, and DOX was loaded onto the NPs to investigate its loading and release.

First, SiO_2_ NPs with diameters of 178 ± 6.1 nm were synthesized by the well-known Stöber method [26,27]. The SiO_2_ NPs were then functionalized with (3-mercaptopropyl)trimethoxysilane (MPTS) to introduce thiol groups, which have high affinity to Ag NPs. To allow the stable and dense immobilization of *β*-CD derivatives, Ag NPs were embedded on the SiO_2_ NP surface by the reduction of silver nitrate with octylamine. Three kinds of ligands (cysteinyl-*β*-CD, EDA-*β*-CD, and methoxypoly(ethylene glycol)sulfhydryl [m-PEG-SH]), prepared following previously reported methods [25,28], were added after the synthesis of SiO_2_@Ag NPs. Cysteinyl-*β*-CD and EDA-*β*-CD (Figure 1b) were selected as functionalized *β*-CDs because they are known to effectively capture DOX and the thiol group of cysteinyl-*β*-CD and diamine of EDA-*β*-CD have strong affinity to metal surfaces [23].

**Figure 2:**
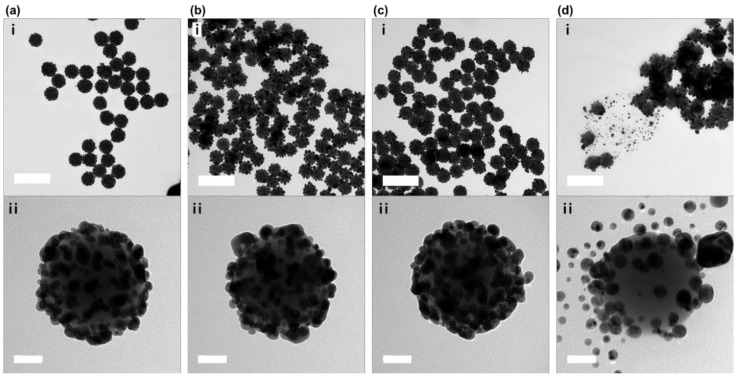
TEM images of SiO_2_@Ag NPs immobilized with three kinds of ligands. (**a**) Bare SiO_2_@Ag NPs, (**b**) SiO_2_@Ag@cysteinyl-*β*-CD NPs, (**c**) SiO_2_@Ag@EDA-*β*-CD NPs, and (**d**) SiO_2_@Ag-PEG NPs. (i) Low-magnification TEM images of the overall morphology of NPs after the addition of ligands. (ii) High-magnification TEM images of single NPs. Scale bars, (i) 500 nm and (ii) 50 nm.

**Figure 3:**
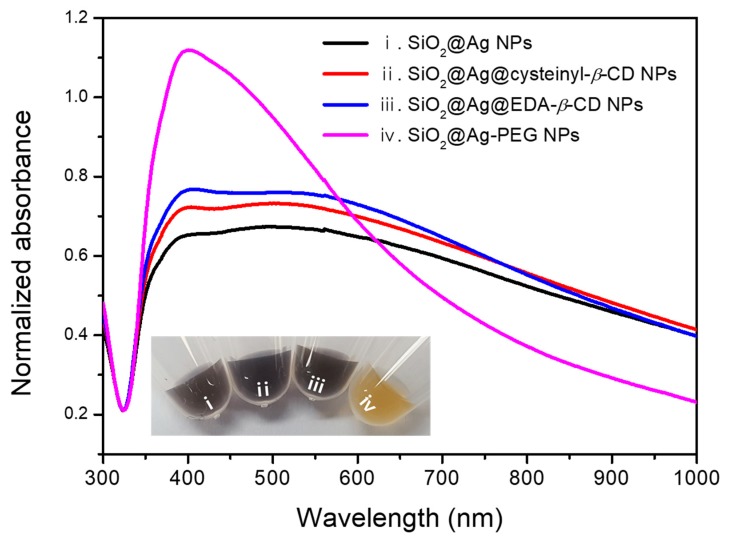
UV-Vis spectra for SiO_2_@Ag NPs, SiO_2_@Ag@cysteinyl-*β*-CD NPs, SiO_2_@Ag@EDA-*β*-CD NPs, and SiO_2_@Ag-PEG NPs. (i) SiO_2_@Ag NPs, (ii) SiO_2_@Ag@cysteinyl-*β*-CD NPs, (iii) SiO_2_@Ag@EDA-*β*-CD NPs, and (iv) SiO_2_@Ag-PEG NPs. (Inset: Photograph of synthesized NP solutions using four kinds of ligands, showing the color change).

To confirm the shape of the produced NPs, transmission electron microscopy (TEM) images were recorded. As shown in Figure 2a, uniform SiO_2_@Ag NPs were synthesized, and Ag NPs with diameters of 16 ± 7 nm were densely immobilized on the SiO_2_ surface following the reduction reaction. Following coating with cysteinyl-*β*-CD or EDA-*β*-CD, the structure of the SiO_2_@Ag NPs was maintained, as shown in Figure 2b,c. However, when the m-PEG-SH solution was used as a ligand, the Ag NPs detached from the SiO_2_ NPs, as shown in Figure 2d. The ultraviolet-visible (UV-Vis) spectra for SiO_2_@Ag@cysteinyl-*β*-CD NPs and SiO_2_@Ag@EDA-*β*-CD NPs were similar to that for SiO_2_@Ag NPs, which absorbed a broad wavelength range from 395 nm to 1000 nm, as shown in Figure 3. The UV-Vis spectrum for SiO_2_@Ag-PEG NPs, which had a peak at 401 nm, was narrower than that for SiO_2_@Ag NPs and similar to that for Ag NPs [29]. In addition, the solution color of SiO_2_@Ag NPs incorporated with *β*-CDs was not significantly different from that of the SiO_2_@Ag NPs, while the solution of SiO_2_@Ag-PEG NPs turned yellow. The introduction of m-PEG-SH might cause Ag NPs to detach from SiO_2_ NPs, and the synthesis of the assembled structure is not easy to control. Therefore, among the three ligands immobilized on nanostructures, the SiO_2_@Ag@cysteinyl-*β*-CD NPs and SiO_2_@Ag@EDA-*β*-CD NPs were used to further analyze DOX loading and release.

**Figure 4:**
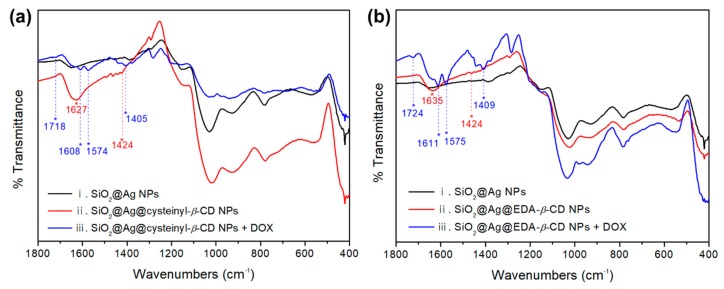
FTIR spectra confirming the introduction of cysteinyl-*β*-CD, EDA-*β*-CD, and DOX on SiO_2_@Ag NPs. (**a**) Immobilized cysteinyl-*β*-CD (N–H bending vibration at 1627 cm^−1^, CH_2_ bending vibration at 1424 cm^−1^, red) on the SiO_2_@Ag NPs and DOX (ketone group stretching at 1718 cm^−1^, C=C stretching vibration at 1608 cm^−1^ and 1574 cm^−1^, and methyl group bending at 1403 cm^−1^, blue) on SiO_2_@Ag@cysteinyl-*β*-CD NPs. (**b**) Immobilized EDA-*β*-CD (N–H bending vibration at 1635 cm^−1^, CH_2_ bending vibration at 1424 cm^−1^, red) on SiO_2_@Ag NPs and DOX (ketone group stretching at 1724 cm^−1^, C=C stretching vibration at 1611 cm^−1^ and 1575 cm^−1^, methyl group bending at 1409 cm^−1^, blue) on SiO_2_@Ag@EDA-*β*-CD NPs. (i) SiO_2_@Ag NPs, (ii) SiO_2_@Ag@cysteinyl-*β*-CD NPs and SiO_2_@Ag@EDA-*β*-CD NPs, (iii) NPs after loading DOX onto SiO_2_@Ag@cysteinyl-*β*-CD NPs and SiO_2_@Ag@EDA-*β*-CD NPs.

To confirm that the SiO_2_@Ag NPs were successfully coated with *β*-CDs and loaded with DOX, attenuated total reflection-Fourier transform infrared (ATR-FTIR) spectra of the synthesized NPs were recorded after each step, as shown in Figure 4 (see also Figure S1). To compare the FTIR spectra of our synthesized materials, we normalized the signal of our material at ~3800 cm^−1^ (background signal). The IR spectra of the SiO_2_@Ag NPs coated with two types of *β*-CDs were similar to that of the SiO_2_@Ag NPs; however, two observations corroborated that *β*-CDs adhered to the SiO_2_@Ag NPs. The IR spectra of the two kinds of *β*-CDs are shown in Figure S2a. The intensity of the band at 1627 cm^−1^ in the IR spectrum for SiO_2_@Ag@cysteinyl-*β*-CD NPs increased due to the N–H bending vibration of *β*-CD derivatives, as shown in Figure 4a. In addition, the intensity of the band at 1635 cm^−1^, which represents the N–H bending vibration of *β*-CD derivatives, was different from that of SiO_2_@Ag@EDA-*β*-CD NPs, as shown in Figure 4b. In particular, the peak around 1000 cm^−1^ is larger than that in the other spectra. This peak is attributed to bonds in the SiO_2_ NPs such as Si–O–Si and Si–OH. However, the peak intensity decreases after modification with DOX. In addition, when the cysteinyl-*β*-CD was introduced onto the SiO_2_@Ag surface, some part of the Ag NPs could detach or move to another Ag or thiol group on SiO_2_, as shown in Figure 2b. The thiol group included in cysteinyl-*β*-CD has a higher affinity to Ag NPs than the amine group. As a result, the surface of SiO_2_@Ag@cysteinyl-*β*-CD NPs exhibits more SiO_2_, and the FTIR spectra could be larger than the other spectra. This observation can be taken as evidence that DOX was loaded onto the surface of the NPs.

A band at 1424 cm^−1^ was assigned to the CH_2_ bending vibration from *β*-CD derivatives (Figure 4ii). Although a CH_2_ group was included in the thiol-functionalized SiO_2_@Ag NPs, CH_2_ bending does not appear in Figure 4i. This is presumably because MPTS is covered with Ag NPs [30]. After loading DOX onto SiO_2_@Ag@cysteinyl-*β*-CD, new bands at 1608 cm^−1^ and 1574 cm^−1^, which were assigned to the aromatic C=C stretching vibration in Figure 4a, clearly indicate the presence of DOX. Moreover, bands at 1718 cm^−1^ and 1403 cm^−1^ appeared, which were assigned to ketone group stretching and methyl group bending vibrations, respectively. The IR spectra for SiO_2_@Ag@EDA-*β*-CD NPs with DOX showed aromatic C=C stretching vibration bands at 1611 cm^−1^ and 1575 cm^−1^. Additionally, ketone group stretching and methyl group bending vibration bands of DOX at 1724 cm^−1^ and 1409 cm^−1^, respectively, were detected. The IR spectra of DOX is shown in Figure S2b. Thus, we confirmed that the *β*-CDs were immobilized on the SiO_2_@Ag NPs, and DOX was loaded onto the NPs.

To evaluate the amount of DOX loaded onto each NP, a DOX solution of 50 µmol/mL (44 µg/mL) was separately added to 1 mg/mL solutions of SiO_2_@Ag@cysteinyl-*β*-CD NP and SiO_2_@Ag@EDA-*β*-CD NP. After vortexing for 12 h to mix the solutions, the supernatant was separated by centrifugation at 13,000 rpm for 15 min.

**Figure 5:**
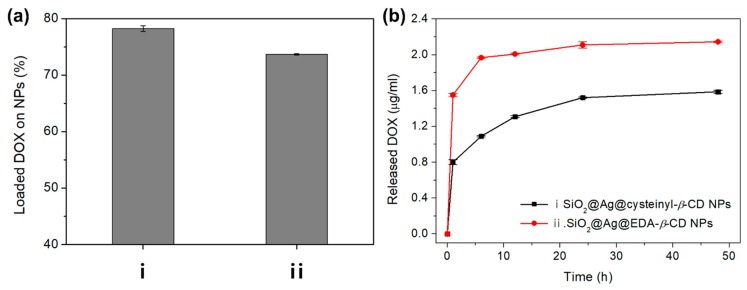
(**a**) The percentage of DOX loaded onto each NP; (i) SiO_2_@Ag@cysteinyl-*β*-CD NPs and (ii) SiO_2_@Ag@EDA-*β*-CD NPs after adding a DOX solution (44 µg/mL) to the NPs. (**b**) The quantity of DOX released from the NPs at room temperature over 48 h. DOX was released more slowly from SiO_2_@Ag@cysteinyl-*β*-CD NPs.

The absorbance of the supernatant was measured at 483 nm, and a DOX calibration curve (Figure S2) was used to evaluate the quantity of DOX loaded onto the synthesized NPs. Figure 5a shows the percentage of DOX loaded onto two kinds of synthesized NPs. The quantity of DOX loaded onto SiO_2_@Ag@cysteinyl-*β*-CD NPs and SiO_2_@Ag@EDA-*β*-CD NPs were 34.4 µg/mg and 32.3 µg/mg, corresponding to 78.2 and 73.6% of the initial DOX, respectively. Thus, slightly more DOX was loaded onto SiO_2_@Ag@cysteinyl-*β*-CD NPs than onto SiO_2_@Ag@EDA-*β*-CD NPs. According to Hassan et al., *β*-CD is a basket-shaped oligosaccharide with a thinner and broader ring’s edge [14]. The exterior part of *β*-CD is hydrophilic, and its internal cavity is relatively non-polar. Due to this construction, non-polar guest can be encapsulated by *β*-CD to form the inclusion complex between CDs and guest by generating hydrogen bonding, hydrophobic interaction, van der Waals interaction. In the same way, DOX can interacts with *β*-CD to generate the supramolecular complex by host-guest inclusion. However, it is not easy to explain the mechanism in which DOX were loaded and released with different rates in SiO_2_@Ag@cysteinyl-*β*-CD NPs and SiO_2_@Ag@EDA-*β*-CD NPs. However, the loading of DOX depends on types of the functional group of *β*-CD previously reported by our group. Ethylenediamine *β*-CD derivative can captured various flavonoids [25]. We believed that not only cavity but also the exterior part of *β*-CD can interact selectively with DOX.

To confirm the release behavior, the DOX release from each NP was monitored over time by measuring the UV-Vis absorbance of the supernatant after 1, 6, 12, 24, and 48 h at room temperature. The supernatant was collected at each time point, and then, a new solution was added for the next release step. The results are shown as the accumulated values of released DOX, as calculated using the DOX calibration curve.

Figure 5b shows the release profiles of the two kinds of NPs as a function of time. The SiO_2_@Ag@EDA-*β*-CD NPs released more DOX than the SiO_2_@Ag@cysteinyl-*β*-CD NPs during the early stage of release as well as more DOX overall than the SiO_2_@Ag@cysteinyl-*β*-CD NPs, which released the lowest levels DOX throughout the release process. Over 48 h, the SiO_2_@Ag@EDA-*β*-CD NPs and SiO_2_@Ag@cysteinyl-*β*-CD NPs released 2.15 µg (7.40%) and 1.57 µg (5.48%) of DOX, respectively. The percentage of released DOX is slightly lower than the reported value of ~10% [31], but the treatment concentration of DOX was lower than those of previous studies [32,33] to avoid side effects. The release behavior of DOX from *β*-CD was reported by Viale et al. [34]. These results reveal the release kinetics of *β*-CD with an amine group, i.e., it releases DOX more slowly when it is loaded onto cationic oligomeric *β*-CD. This released quantity of DOX from SiO_2_@Ag@EDA-*β*-CD NPs could be explained by the positive charge owing to NH_2_ at neutral pH.

Figure 5 shows that the highest amount of DOX was introduced onto the SiO_2_@Ag@cysteinyl-*β*-CD NPs, and less DOX was released by the SiO_2_@Ag@cysteinyl-*β*-CD NPs. Thus, we believe that DOX is captured by cysteinyl-*β*-CD in the SiO_2_@Ag@cysteinyl-*β*-CD NPs. These results indicate that the loading and release of DOX on NPs depends on the kind of *β*-CD ligand. Furthermore, the observed release behavior indicates that among the two types of NPs examined, the SiO_2_@Ag@cysteinyl-*β*-CD NPs could be a good candidate for capturing and sequestering DOX in drug delivery systems.

**Figure 6:**
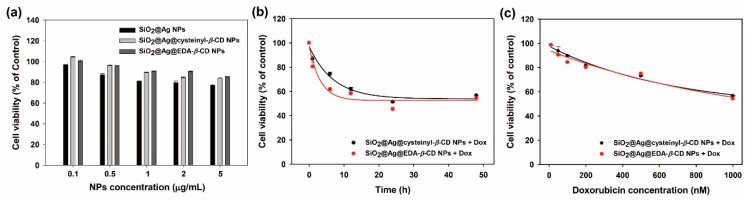
Assessment of cytotoxicity in cancer cells treated with Ag-embedded SiO_2_ nanocarriers. (**a**) MCF-7 cancer cells were treated with each type of NP at increasing concentrations (0.1–5 µg/mL) for 48 h. The cell viability was measured using the WST-1 assay. (**b**) Cell viability after treatment with cysteinyl-*β*-CD and EDA-*β*-CD loaded with 1 µM DOX with increasing incubation time (1, 6, 12, 24 and 48 h). The cytotoxicity rate was obtained by fitting with an exponential equation (lines). (**c**) Cell viability after treatment with various concentrations of DOX-loaded cysteinyl-*β*-CD and EDA-*β*-CD NPs for 48 h. The decrease in cell viability with increasing DOX was fit to the hyperbolic equation (lines).

To assess cysteinyl-*β*-CD and EDA-*β*-CD NPs as an anticancer drug carrier, the cell viability was measured in cancer cells treated with cysteinyl-*β*-CD and EDA-*β*-CD with or without DOX (Figure 6). The cytotoxic effects of SiO_2_@Ag, SiO_2_@Ag@cysteinyl-*β*-CD, and SiO_2_@Ag@EDA-*β*-CD NPs on breast cancer cells (MCF-7 cells) were negligible up to a concentration of 5 µg/mL (Figure 6a). This result indicates that our Ag-embedded SiO_2_ NPs were biocompatible and not significantly cytotoxic. In contrast, when DOX-loaded cysteinyl-*β*-CD and EDA-*β*-CD nanocarriers were incubated with MCF-7 cells at 37 °C with increasing incubation time, the viability of MCF-7 cells significantly decreased from 1 h to 48 h (Figure 6b). The rate of cytotoxicity with DOX-loaded SiO_2_@Ag@EDA-*β*-CD was faster than that of DOX-loaded SiO_2_@Ag@cysteinyl-*β*-CD. In addition, the cell viability of cancer cells dropped sharply to ~60% after 12 h if incubation with both DOX-loaded cysteinyl-*β*-CD and EDA-*β*-CD nanocarriers, which was maintained until 48 h. This result was consistent with the DOX release time (Figure 5b) and demonstrated that DOX was completely released from the nanocarriers after 12 h. The cytotoxicity of DOX loaded onto the nanocarriers was also investigated, as shown in Figure 6c. The cell viability decreased with increasing DOX concentrations. At low DOX concentrations (<200 nM), the cell viability was ~80%, which decreased to 60% at 1000 nM. The concentration of DOX needed to attain 50% cell viability (GI50) was calculated by fitting with a hyperbolic equation, providing GI50 values of 1.323 µM and 1.154 µM for SiO_2_@Ag@cysteinyl-*β*-CD and SiO_2_@Ag@EDA-*β*-CD, respectively.

## 3. Materials and Methods

### 3.1. Materials

To synthesize the NPs, tetraethyl orthosilicate (TEOS), ethylene glycol, polyvinylpyrrolidone (PVP, Mw ≈ 40,000), silver nitrate (AgNO_3_, 99.99%), octylamine, and MPTS were purchased from Sigma-Aldrich (St. Louis, MO, USA) and used without any purification. Ethyl alcohol (EtOH) and aqueous ammonium hydroxide (NH_4_OH, 27%) were purchased from Daejung (Siheung, Korea). Designed cysteinyl-*β*-CD and EDA-*β*-CD were obtained from the Microbial Carbohydrate Resource Bank (MCRB) at Konkuk University, Korea [25,28], and m-PEG-SH (M.W. 5000) was purchased from Sunbio (Anyang, Korea). Deionized (DI) water was used in all experiments.

### 3.2. Synthesis of SiO_2_@Ag NPs

The SiO_2_@Ag NPs were synthesized according to a previously reported method [24]. SiO_2_ NPs were prepared by a modified Stöber method [26] using 40 mL of 99.9% EtOH, 3 mL of NH_4_OH, and 1.6 mL of TEOS. The solution was stirred vigorously for 20 h at room temperature and then washed with 95% EtOH three times. To synthesize the embedded Ag NPs, PVP in 25 mL of ethylene glycol was mixed with the thiol-functionalized silica solution (30 mg/mL). AgNO_3_ in ethylene glycol and octylamine were added in sequence. After a reaction time of 1 h, the sample was washed with 95% EtOH several times. To obtain a 10 mg/mL SiO_2_@Ag NP solution, it was dispersed in 3 mL of absolute EtOH. The NPs were examined with an energy-filtering transmission electron microscope (LIBRA 120; Carl Zeiss, Oberkochen, Germany) operated at an accelerating voltage of 120 kV.

### 3.3. Preparation of SiO_2_@Ag@cysteinyl-β-CD NPs, SiO_2_@Ag@EDA-β-CD NPs, and SiO_2_@Ag-PEG NPs

To prepare SiO_2_@Ag@cysteinyl-*β*-CD NPs, the cysteinyl-*β*-CD solution (1 mmol in DI water) was supplemented with 1 mg of SiO_2_@Ag NPs. The mixture was vortexed vigorously for 12 h at 25 °C. The suspension was washed one time with 95% EtOH by centrifugation and dispersed in 1 mL of absolute EtOH. The SiO_2_@Ag@EDA-*β*-CD NPs were synthesized using the same method as that for SiO_2_@Ag@cysteinyl-*β*-CD NPs. To synthesize SiO_2_@Ag-PEG NPs, m-PEG-SH solution (1 mmol in DI water) was added to 1 mg of SiO_2_@Ag NPs. The mixture was vortexed vigorously for 12 h at room temperature and then washed once with 95% EtOH by centrifugation. Finally, it was dispersed in 99% EtOH.

### 3.4. Loading of DOX on SiO_2_@Ag NPs with Ligands (β-CD Derivatives and PEG)

A DOX solution was added to SiO_2_@Ag@*β*-CD derivative NP and SiO_2_@Ag-PEG NP (1 mg/mL) solutions. The concentration of DOX dispersed in DI water was 50 µmol/mL. The mixture was vigorously shaken at room temperature for 12 h in the dark. Free DOX was removed by centrifugation at 13,000 rpm for 15 min. To determine the amount of DOX introduced, a calibration curve was obtained based on absorbance at 483 nm using various concentrations of DOX in 50% EtOH, as shown in Figure S3.

### 3.5. DOX Release

DOX release was observed at room temperature without additional or external factors. To monitor the release of DOX, the supernatant of DOX-loaded SiO_2_@Ag@*β*-CD derivative NPs was measured at 1, 6, 12, 24, and 48 h. The supernatant of each NP was harvested after centrifugation at 13,000 rpm for 15 min. To measure the released amount of DOX, the absorbance of the supernatant was measured at 483 nm using a UV spectrophotometer (OPTIZEN POP; Mecasys, Daejeon, Korea).

### 3.6. Cell Culture and Cell Viability Assay

The cells used in this study were MCF-7 human breast cancer cells purchased from ATCC (American Type Culture Collection; code no ATCC^®^ HTB-22^TM^). MCF-7 cells were cultured in DMEM (Dulbecco’s Modified Eagle Medium) culture medium (HyClone Laboratories, Logan, UT, USA) supplemented with 10% fetal bovine serum (HyClone Laboratories) and 1% of penicillin/streptomycin (Welgene, Daegu, Korea). MCF-7 cells were seeded onto 96-well plates at a density of 5.0 × 10^3^ cells/well and incubated at 37 °C for 24 h. The WST-1 assay was then performed according to the manufacturer’s instructions 48 h after treatment with NPs of DOX-loaded NPs at the indicated concentrations. The absorbance was measured by VICTOR X3 multi-label plate reader (PerkinElmer, Waltham, MA, USA) at 450 nm.

## 4. Conclusions

Functionalized *β*-CD derivatives, namely, cysteinyl-*β*-CD and EDA-*β*-CD, were successfully immobilized on SiO_2_@Ag NPs to load DOX. The percentages of DOX introduced onto each NP were similar; however, the release behavior differed. In comparison to SiO_2_@Ag@EDA-*β*-CD NPs, the SiO_2_@Ag@cysteinyl-*β*-CD NPs captured relatively more and released less DOX. Moreover, the cell viability was decreased by increasing the concentration of NPs with DOX. These features indicate that *β*-CDs, in particular SiO_2_@Ag@cysteinyl-*β*-CD NPs, are useful candidate materials for drug capture and show promise for the development of bioapplications and nanomedicine, with particular potential for drug delivery systems.

## Supplementary Materials

Supplementary materials can be found at http://www.mdpi.com/1422-0067/20/2/315/s1.
