# Supplementary material for: Functionalized β-Cyclodextrin Immobilized on Ag-Embedded Silica Nanoparticles as a Drug Carrier
Source: Int J Mol Sci. 2019 Jan 14;20(2):315. doi: 10.3390/ijms20020315 (PMC6359520; doi:10.3390/ijms20020315)
Supplement: Supplementary file 1 [file ijms-20-00315-s001.pdf]

Supplementary Materials

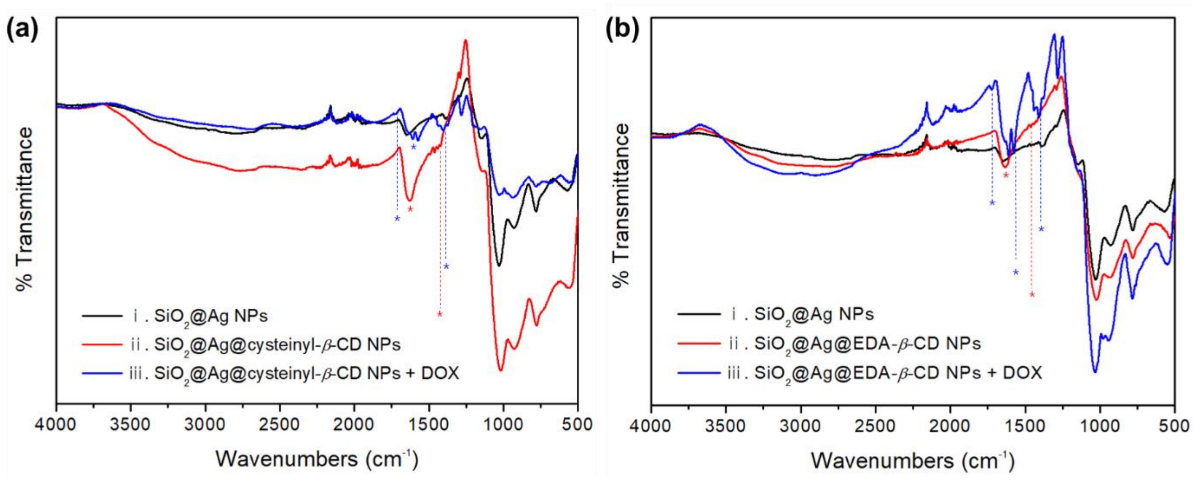

Figure S1. Broad-range FTIR spectra (from 4000 to 500 cm^{-1}) confirming the introduction of cysteinyl-*β*-CD, EDA-*β*-CD, and DOX on modified SiO_2@Ag NPs. FTIR spectra of i) SiO_2@Ag NPs, ii) SiO_2@Ag@cysteinyl-*β*-CD NPs and iii) SiO_2@Ag@cysteinyl-*β*-CD NPs (**a**) before and (**b**) after the introduction of DOX. Normalized by the signal of the material at ~3800 cm^{-1}.

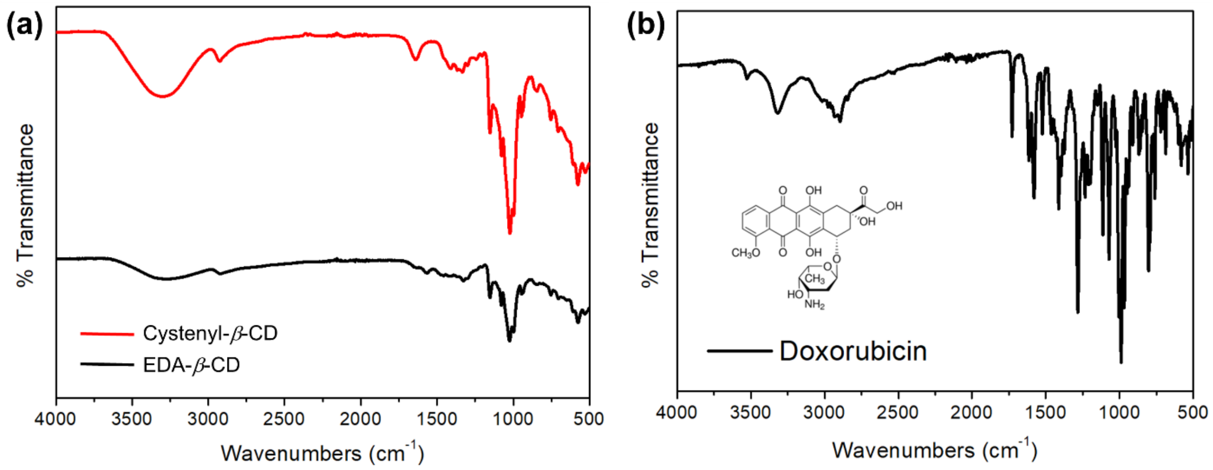

Figure S2. FTIR spectrum of (**a**) cysteinyl-*β*-CD and EDA-*β*-CD, (**b**) DOX. (Inset: The chemical structure of DOX).

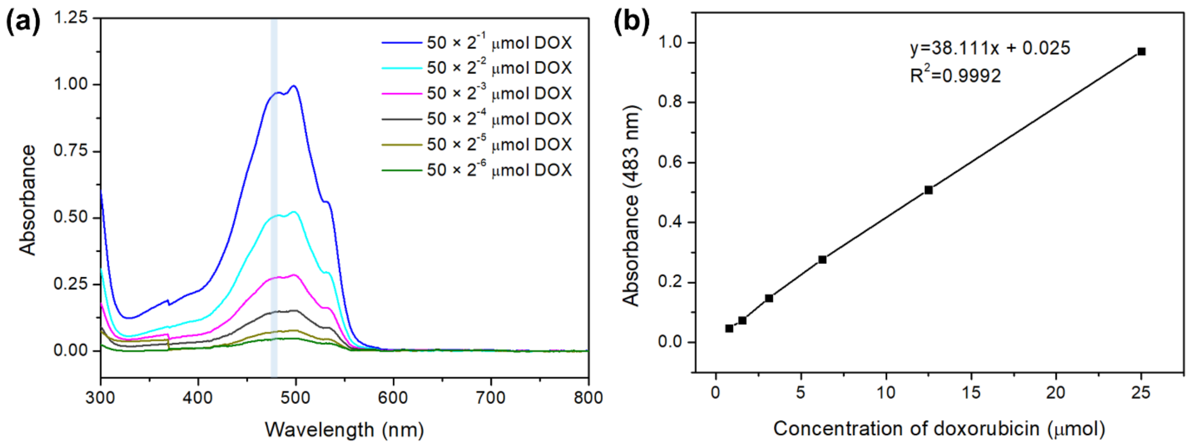

Figure S3. (**a**) UV-Vis spectra of doxorubicin (DOX). The wavelength of 483 nm was marked with vertical line inside of the graph. (**b**) Calibration curve of DOX aqueous solution as a function of concentration.
